# Inhalation Exposure to 2,4-Dichlorophenoxyacetic Acid Causes Tracheal Damage—A Study in Rats

**DOI:** 10.3390/toxics14040271

**Published:** 2026-03-24

**Authors:** Anna Carolina Ferretti Wisenfad, Isabela Vieira Duran, Luciana Shiraichi Barga, Gisele Alborghetti Nai

**Affiliations:** 1 Faculty of Medicine of Presidente Prudente, Universidade do Oeste Paulista (UNOESTE), Presidente Prudente 19050-680, SP, Brazil; annacafw13@gmail.com; 2 Faculty of Veterinary Medicine of Presidente Prudente, Universidade do Oeste Paulista (UNOESTE), Presidente Prudente 19050-680, SP, Brazil; isaduran.1006@gmail.com; 3 Graduate Program in Health Sciences, Universidade do Oeste Paulista (UNOESTE), Presidente Prudente 11441-225, SP, Brazil; lushiraichi@gmail.com; 4 Faculty of Medicine of Guarujá, Universidade do Oeste Paulista (UNOESTE), Guarujá 11441-225, SP, Brazil; 5 Department of Pathology, Universidade do Oeste Paulista (UNOESTE), Presidente Prudente 19050-680, SP, Brazil

**Keywords:** herbicides, tracheal diseases, inflammation, pesticide exposure, occupational exposure

## Abstract

Exposure to 2,4-dichlorophenoxyacetic acid (2,4-D) occurs mainly by inhalation. Studies indicate that respiratory allergic reactions are induced by this pesticide. This study analyzed the effects of chronic inhalation exposure to 2,4-D in the trachea of rats. We exposed the animals during six months to three different concentrations of 2,4-D used for crop spraying. Animals exposed to low concentrations had an increase in the mast cells count, showing that this herbicide can cause allergic reactions in the airways. The tracheal epithelium thickness, the nuclear area and number of nucleolar organizing regions increased proportionally to the exposure concentration and in correlation with each other. These histological tissue changes correspond to epithelial hypertrophy and increased cell activity and multiplication, and show an adaptation to tissue damage caused by 2,4-D. There was a tendency for inversion in the mucus pattern to acid and a decrease in goblet cells in the groups exposed to 2,4-D, which alters the protective mechanisms of the respiratory tract. The 2,4-D induced adaptations in the tracheal epithelium associated with an increase in herbicide concentrations, which may compromise mucociliary function and predispose the epithelium to additional lesions over time. Therefore, it should be used with appropriate respiratory protection equipment to avoid injury.

## 1. Introduction

2,4-dichlorophenoxyacetic acid (2,4-D) is a synthetic auxin used for the selective control of broadleaf plants, sparing grasses and other narrowleaf crops [1]. Despite its effectiveness in eradicating unwanted plants, numerous cases of poisoning and toxicity associated with 2,4-D exposure have been reported, raising significant concerns about its safety for both human health and the environment [2].

Human exposure to 2,4-D, particularly to its salts or esters, occurs through ingestion, inhalation or dermal absorption. This could result from occupational exposure during the production or application of the product, as well as from contact with 2,4-D residues present in food, water, air, or soil. One form of exposure to 2,4-D is its presence in the external air, resulting from spray drift during application, volatilization of applied products or even suspension of its particles [1,2,3,4].

Exposure to 2,4-D can cause acute symptoms such as coughing, burning, dizziness, loss of muscle coordination, nausea, diarrhea, and vomiting. In addition, studies have identified gene damage related to the stress response, cell cycle control, DNA repair, and immunity associated with herbicide exposure, indicating possible chronic effects on the body [1,4,5]. However, it is important to highlight that several studies indicate the lack of conclusive data in humans that would allow us to affirm a causal relationship between exposure to 2,4-D and these changes [1,6,7,8].

Owing to these uncertainties, the classification of 2,4-D’s safety varies among countries. In Canada and the USA, it is not considered harmful as long as safety measures are respected. To mitigate potential toxic effects, reference doses in Europe were updated and have since been similar to those of the Brazilian National Health Surveillance Agency (ANVISA) [7,8,9]. In the study conducted by the Agricultural Health Study, several respiratory outcomes, including wheezing, farmer pneumonitis, chronic bronchitis, asthma, and rhinitis, were investigated in humans exposed to 2,4-D. The lack of a clear dose–response relationship and the multiple coexposures make it difficult to determine the specific effects of 2,4-D [8]. Additionally, research on veterans exposed to Agent Orange—a defoliant containing 2,4-D as a primary component—during the Vietnam War (1962–1971) showed a significant increase in noncancerous respiratory conditions and a higher risk of mortality [10].

The respiratory system is in contact with the external environment because of the flow of air within it. Its mucosa serves as an interface between the internal environment and inhaled air, protecting the body against impurities in the air through defense mechanisms present mainly in the conducting portion, of which the trachea is a component. Thus, exposure to toxic chemical compounds, such as 2,4-D, can have negative effects on respiratory health by affecting the defense mechanisms present in the respiratory mucosa [6,11].

Although studies have associated 2,4-D with respiratory problems, the relationship between 2,4-D exposure and its specific consequences in the trachea has rarely been explored. Research on the toxicity of chemical compounds in the trachea has revealed that harmful effects can compromise the function of the respiratory system and increase the risk of pulmonary and tracheobronchial infections [12].

We evaluated the toxic effects of chronic exposure to 2,4-D via inhalation on the trachea of rats under conditions and concentrations similar to those used for human exposure.

## 2. Materials and Methods

### 2.1. Chemical

The herbicide used was 2,4-D (Nortox^®^ S.A., Arapongas, Paraná, Brazil), with the following composition: dimethylamine salt of 2,4-dichlorophenoxyacetic acid: 806 g/L (80.6% m/v), acid equivalent of 2,4-D: 670 g/L (67.0% m/v) and inert ingredients: 424 g/L (42.4% m/v).

### 2.2. Experimental Animals

The sample size for comparing scores for 4 groups was calculated using the “pwr” package, available in the R program (version R-4.5.2 for Windows). We used these parameters: test power = 80%; significance level = 5%; number of groups to be compared = 4; effect size (Cohen’s D) = 0.50. Thus, at least 8 animals per group were necessary. To adapt them to a possible non-parametric distribution, we added 15% more animals, and the final number was 10 per group.

Forty adult male rats (*Rattus norvegicus* Wistar) weighing between 200 and 250 g provided by the Universidade do Oeste Paulista (UNOESTE) Central Bioterium were used. They were placed in plastic cages (three to four animals) in a room with temperature and light-dark control.

The animals were distributed by randomization divided by drawing of lots into four groups (*n* = 10): C—control: exposed to distilled water; L[ ]—low-concentration: exposed to the pesticide with 3.71 × 10^−3^ g of active ingredient per hectare (g.a.i./ha), corresponding to 20.69 parts per million (ppm) of 2,4-D; M[ ]—medium-concentration: exposed to the pesticide with 6.19 × 10^−3^ g.a.i./ha, corresponding to 34.63 ppm of 2,4-D; H[ ]—high-concentration: exposed to the pesticide with 9.28 × 10^−3^ g.a.i./ha, corresponding to 51.66 ppm of 2,4-D.

The animals have been treated humanely according to institutional guidelines and with the internationally accepted principles for laboratory animal use and care as found in the international guidelines, with due consideration to the alleviation of distress and discomfort or pain.

### 2.3. Exposure Protocol for the Herbicide 2,4-D

Three different concentrations of 2,4-D, recommended in the manufacturer’s leaflet for controlling various weeds in different crops, were used in this study. These concentrations were adjusted to the box area to simulate environmental exposure and were diluted in 10 mL of distilled water for nebulization [13].

For the exposure process, two polypropylene boxes (32 × 24 × 32 cm) were connected to a nebulizer (Pulmosonic Star^®^, Soniclear Ind. Com. Imp. e Exp. Ltda., São Paulo, Brazil). The exposure lasted approximately 15 min, the time required for complete nebulization of the solution [13].

To replicate occupational exposure, the animals were subjected to the nebulized solution five days during the week. After six months, they were euthanized using sodium thiopental (Syntec, Henderson, NC, USA) at a dose of 100 mg/kg via intraperitoneal injection. Death was confirmed by the absence of respiratory movements, heartbeats, and reflexes [14].

### 2.4. Pathological Analysis

The trachea was collected and fixed in 10% buffered formalin (Cinética Indústria Química, São Paulo, Brazil) for 24 h and subsequently sectioned transversally into three areas (proximal, middle and distal) and submitted to histological processing, with paraffin embedding (Dinâmica Reagentes Analíticos, São Paulo, Brazil).

Serial 5 µm sections were obtained and stained via the hematoxylin–eosin (HE) method (Dolles, São Paulo, Brazil), and the following histopathological parameters were analyzed: presence and degree of inflammation; type of inflammatory cells (lymphomononuclear, polymorphonuclear or both); squamous metaplasia; presence and degree of mucosal dysplasia; and neoplasia (benign or malignant) [12]. The pathological analysis was blinded to the treatments.

All images used in the study were acquired using a Leica DM750 optical microscope (Leica Microsystems, Wetzlar, Germany).

#### 2.4.1. Quantification of Mast Cell

Additional 5 µm sections were obtained and subjected to toluidine blue staining (Merck, Darmstadt, Germany) for mast cell identification. Mast cells were counted in 10 high-power fields (HPFs), which corresponded to an area of 1 mm^2^ [15].

#### 2.4.2. Measurement of Tracheal Epithelium Thickness

A histological image of the HE-stained tracheal epithelium per animal was acquired at 400× magnification, and measurements were taken in four areas using the ImageJ software (version 2023) (United States National Institute of Health—NIH—available at https://imagej.net/ij/, accessed on 25 October 2024) [12].

#### 2.4.3. Analysis of the Nucleus Size of Tracheal Epithelial Cells

A histological image of the HE-stained tracheal epithelium per animal at 400× magnification (Leica Microsystems, Germany) was used to measure the area of 50 epithelial nuclei per animal via the elliptical selection and free hand selection tools of ImageJ software (NIH, Bethesda, MD, USA).

#### 2.4.4. Quantification of Goblet Cells and Identification of the Mucus Pattern

Serial sections of 5 µm were obtained and subjected to Periodic acid–Schiff (PAS)-Alcian blue staining (Merck, Darmstadt, Germany), and the goblet cells with acidic mucus and with neutral mucus were counted at 10 HPF.

#### 2.4.5. Analysis of the Presence of Mucus in Goblet Cells

An image of the tracheal epithelium of each animal stained with PAS-Alcian blue (Merck, Darmstadt, Germany) at 400× magnification was used. Mucus quantification in the cytoplasm of goblet cells was performed using the color threshold and RBG measurement tools via ImageJ software (NIH, USA).

#### 2.4.6. AgNOR Staining

Deparaffinized tracheal epithelium sections on slides were incubated in a humid chamber for 40 min at 60 °C in a silver nitrate solution protected from light. The samples were removed from the staining solution when they turned dark brown and were subsequently washed in warm deionized water at 45 °C. They were then passed through a 5% sodium thiosulfate solution for 5 min to remove the reduced silver deposited on the cells. The sections were counterstained with van Gieson (Merck KGaA, Darmstadt, Germany) [16].

Ten AgNOR-stained histological images of the tracheal epithelium per animal at 400× magnification were captured using a Leica DM750 optical microscope (Leica Microsystems, Germany). The nucleolar organizing regions (NORs) were counted in 10 epithelial nuclei/image, totaling 100 nuclei/animal [16].

### 2.5. Statistical Analysis

Statistical analysis was conducted using the Jamovi package v. 2.3 [17]. Histopathological data were evaluated using the likelihood ratio, while quantitative variables were analyzed through analysis of variance (ANOVA), followed by Tukey’s multiple comparison test. All ANOVA assumptions, including normality and homogeneity of variances, were satisfied for all variables. Since the variables presented a normal distribution, Pearson’s correlation was used. The significance level for all tests was 5%.

Cohen’s d was used to assess the effect size. The effect can be considered insignificant (d < 0.19), small (d = 0.20–0.49), medium (d = 0.50–0.79), large (d = 0.80–1.29) or very large (d > 1.30) [18].

## 3. Results

### 3.1. Pathological Analysis

Inflammation is a response of the body to microbiological agents or tissue damage. Although it is a protective response, inflammation is potentially damaging, as in cases of chronic inflammation, resulting in tissue destruction and scarring [19].

Metaplasia is an adaptive cellular change in which a cell type that is more sensitive to an injury is replaced by a more resistant one [19]. Although metaplasia is an adaptive mechanism to an injury, it leads to the loss of natural protective mechanisms; for example, in the case of the respiratory epithelium, the loss of epithelial cilia, which are important for mucus clearance.

2,4-D has limited evidence of carcinogenicity in experimental animal models and is considered possibly carcinogenic to humans (Group 2B) by the International Agency for Research on Cancer (IARC) [1]. Therefore, the constant evaluation of this aspect of their formulations is important.

**Figure 1:**
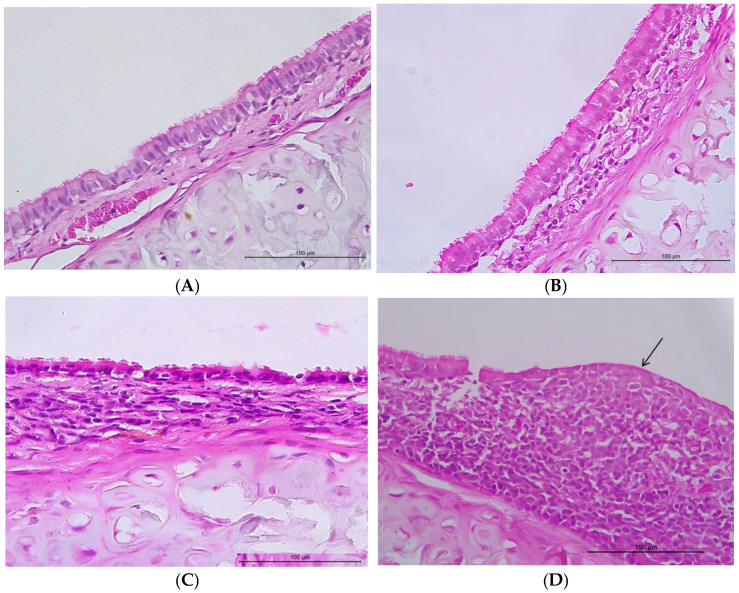
Photomicroscopy of the trachea. (**A**) Tracheal epithelium without alterations (animal from the control group). (**B**) Mild chronic inflammatory process (animal from the group exposed to a low concentration of 2,4-D). (**C**) Moderate chronic inflammatory process (animal from the group exposed to a medium concentration of 2,4-D). (**D**) Intense chronic inflammatory process and area of squamous metaplasia of the epithelium (arrow) (note the loss of cilia and the presence of cuboidal cells in the epithelium) (animal from the group exposed to high concentrations of 2,4-D). Hematoxylin–eosin, 400× magnification (scale bar: 100 µm).

The incidence of inflammation or squamous metaplasia was not different in the 2,4-D groups compared with the control group (*p* > 0.05) (Table 1). Only one animal in the high concentration group presented intense inflammation. The inflammatory infiltrate was lymphoplasmacytic in all cases (Figure 1).

None of the animals evaluated presented dysplastic or neoplastic changes.

Thus, 2,4-D was not found to trigger tissue damage significant enough to stimulate a more pronounced inflammatory process in the tissue or even epithelial metaplasia. The fact that rodents are in close contact with the ground and thus can inhale substances that may damage the respiratory epithelium may explain why we observed some cases of inflammation and metaplasia of the epithelium in the control group. 2,4-D also showed no carcinogenic effect on the respiratory epithelium.

#### 3.1.1. Quantification of Mast Cell

Mast cells are associated with type I (directed against intracellular pathogens), type III (directed against extracellular bacteria and fungi), and type IVb (associated with allergies) hypersensitivity reactions. They are present in tissues that form the body’s major barriers, such as the skin, respiratory and intestinal mucosa, and blood vessels, classic sites of allergic diseases [20].

**Figure 2:**
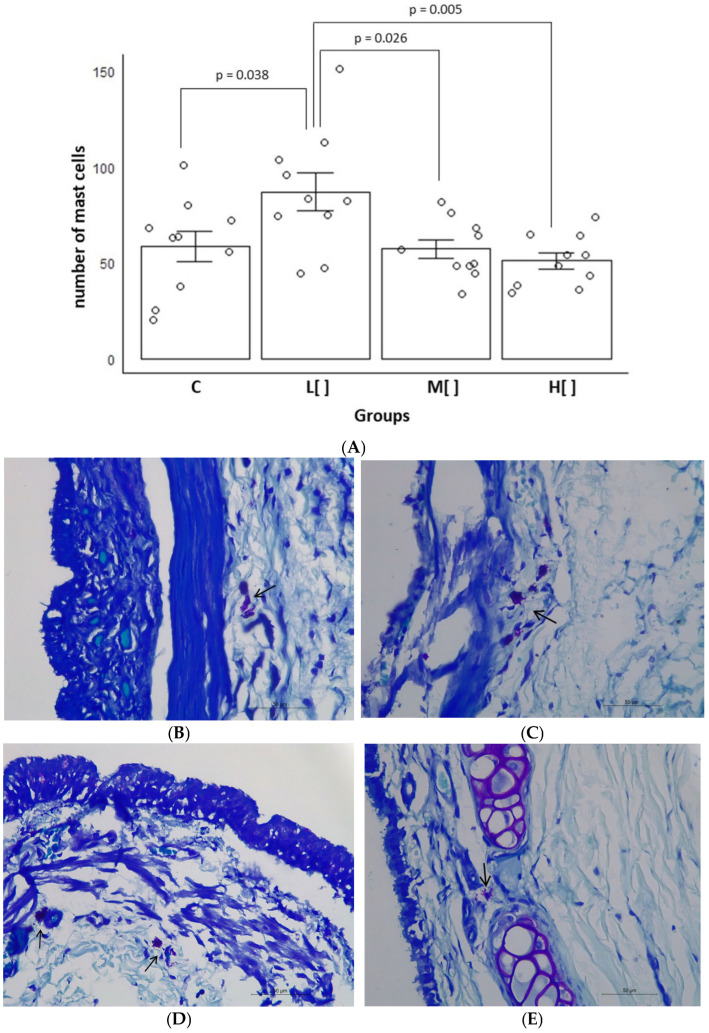
(**A**) Mean and standard error of the number of mast cells (per mm^2^) in the tracheal epithelium according to the study group (*n* = 40). Photomicroscopy of the trachea showing mast cells: (**B**) Small number of mast cells in the tissue (arrow) (animal in the control group). (**C**) Large number of mast cells in the tissue (arrow) (animal exposed to a low concentration of 2,4-D). (**D**) Small number of mast cells in the tissue (arrows) (animals exposed to a medium concentration of 2,4-D). (**E**) Small number of mast cells in the tissue (arrow) (animals exposed to a high concentration of 2,4-D). Toluidine blue, 400× magnification (scale bar: 50 μm). C: control group; L[ ]—group exposed to a low concentration of 2,4-D; M[ ]—group exposed to a medium concentration of 2,4-D; H[ ]—group exposed to a high concentration of 2,4-D.

The animals of the low concentration group had the greatest mast cells count in the tracheal epithelium [mean of 87.0 (standard deviation—SD: 31.38) mast cells/mm^2^] (*p* < 0.05) (Figure 2).

The mast cell count in exposed animals showed a slight difference compared to the control group (Cohen’s d = 0.258412). However, animals exposed to a low concentration of 2,4-D exhibited a large difference in comparison to the control group (d = 1.05161) and the group exposed to a medium concentration (d = 1.276391). Furthermore, the difference between the low-concentration and high-concentration exposure groups was very large (d = 1.564821).

Exposure to low concentrations of 2,4-D resulted in the highest number of mast cells in the tissue, as well as the greatest effect on this parameter, showing that low concentrations of 2,4-D can elicit allergic responses.

#### 3.1.2. Tracheal Epithelial Thickness Measurement

Hypertrophy, hyperplasia, and atrophy are also considered adaptive changes in tissues, where reversible changes occur in the number, size, metabolic activity, or functions of cells in response to changes in their environment. Epithelial cells, classified as stable cells (those that have the ability to undergo mitotic division when stimulated), tend to first undergo hypertrophy (increase in cell volume) and then hyperplasia (increase in the cell number) in response to an overload of demand [19].

**Figure 3:**
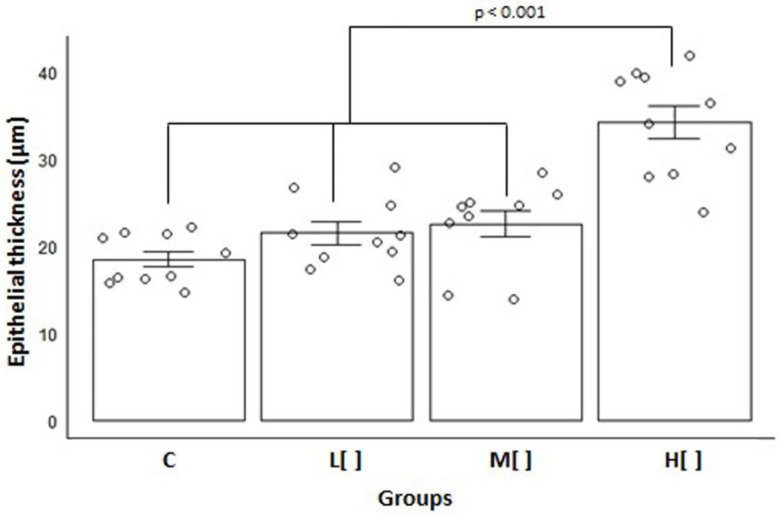
Mean and standard error of tracheal epithelial thickness measurements (in µm) by study group (*n* = 40). C: control group; L[ ]—group exposed to a low concentration of 2,4-D; M[ ]—group exposed to a medium concentration of 2,4-D; H[ ]—group exposed to a high concentration of 2,4-D.

Animals subjected to a high concentration of 2,4-D displayed a significantly greater tracheal epithelial thickness than the other groups [mean = 34.26 μm, SD = 6.11; *p* < 0.001] (Figure 3).

Tracheal epithelial thickness in exposed animals differed substantially from that of the control group (Cohen’s d = 1.321204). The high-concentration exposure group exhibited a very large difference compared to the control group (d = 3.307962), the low-concentration group (d = 2.446866), and the medium-concentration group (d = 2.132782). The low- and medium-concentration exposure groups demonstrated a large difference compared to the control group [(d = 0.850173) and (d = 1.039994), respectively], but only a small difference between them (d = 0.23983).

The increased thickness of the epithelium in animals exposed to high concentrations of 2,4-D shows an overload of demand on the cells, which needed to adapt by undergoing hypertrophy.

#### 3.1.3. Analysis of the Nucleus Size of Tracheal Epithelial Cells

An increase in nuclear volume (nuclear hypertrophy) can occur in association with adaptive changes, when there is increased cellular activity, such as in inflammatory or reparative processes and during the S and G2 phases of interphase, due to the duplication of genetic material (DNA) before cell division [19].

**Figure 4:**
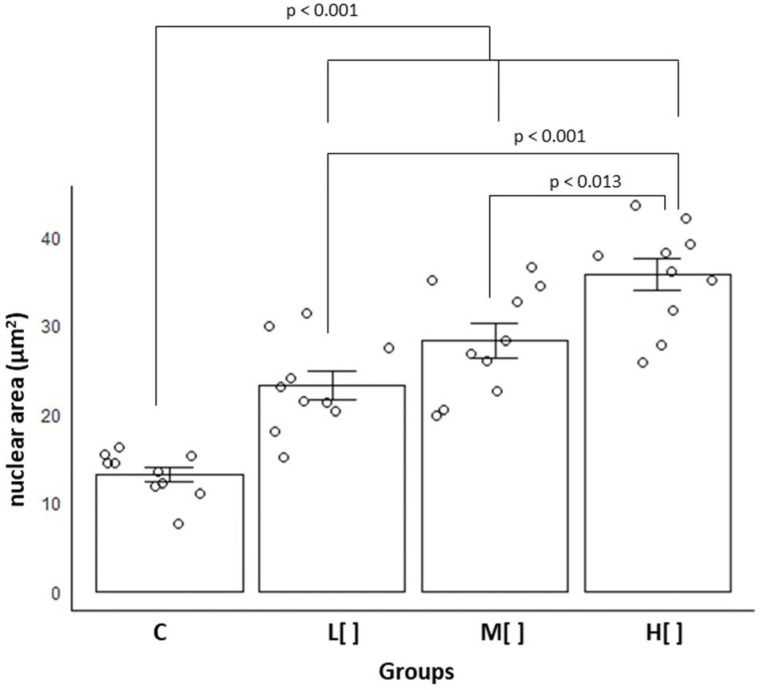
Mean and standard error of the area of the nuclei of tracheal epithelial cells (in µm^2^) by study group (*n* = 40). C: control group; L[ ]—group exposed to a low concentration of 2,4-D; M[ ]—group exposed to a medium concentration of 2,4-D; H[ ]—group exposed to a high concentration of 2,4-D.

Among all groups, animals exposed to the highest concentration of 2,4-D exhibited the greatest mean nuclear area in tracheal epithelial cells [mean = 35.66 µm^2^ (SD = 5.78); *p* < 0.001] (Figure 4).

Exposed animals showed a substantial difference in nuclear area size compared to the control group (Cohen’s d = 2.794365). Specifically, the high-concentration group exhibited a very large difference relative to the control group (d = 5.035774), while the low- and medium-concentration groups showed large differences (d = 2.290134 and d = 1.244838, respectively). Additionally, the low-concentration group differed significantly from the medium-concentration group (d = 0.892123).

The increase in nuclear size associated with exposure to 2,4-D demonstrates that cells exposed to this herbicide exhibit greater cellular activity or are in cell division.

#### 3.1.4. Quantification of Goblet Cells and Mucus Pattern Identification

Goblet cells are responsible for producing mucus, which provides mechanical protection to the respiratory epithelium. A normal number of goblet cells is important for maintaining adequate mucus production. In the respiratory epithelium, these cells predominantly produce neutral mucus [11].

**Figure 5:**
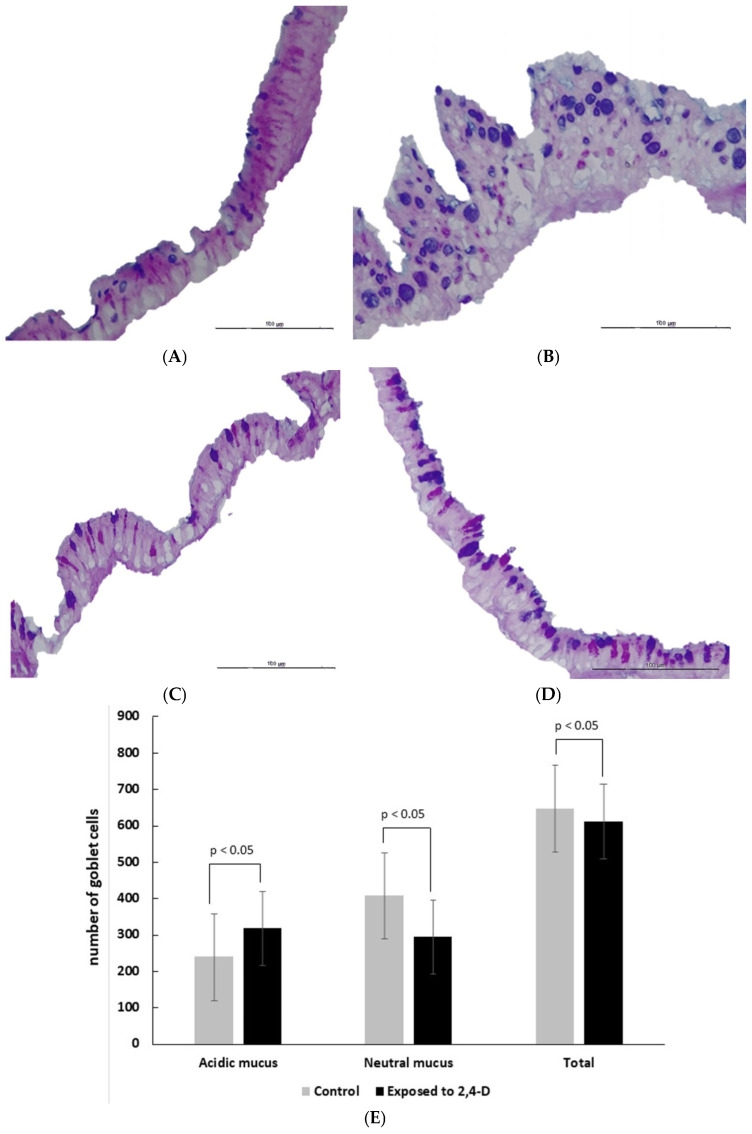
Section of the tracheal epithelium showing goblet cells with neutral mucus (stained pink) and acidic mucus (stained blue) in the cytoplasm. (**A**) Predominance of goblet cells with neutral mucus (animal from the control group). (**B**) Increase in the number of goblet cells and predominance of acidic mucus (animal exposed to low concentrations of 2,4-D). (**C**) A decrease in the number of goblet cells, with a slight predominance of acidic mucus (animals exposed to a medium concentration of 2,4-D). (**D**) Similar proportions of cells with acidic mucus and neutral mucus (animals exposed to a high concentration of 2,4-D). PAS-Alcian blue at 400× magnification (scale bar: 100 μm). (**E**) Mean and standard error of the number of goblet cells (per mm^2^) according to the mucus pattern and exposure to the herbicide 2,4-D.

In the control group, goblet cells predominantly contained neutral mucus, with fewer cells producing acidic mucus. Animals of the low concentration of 2,4-D exhibited the highest number of goblet cells with acidic mucus, whereas those exposed to a medium concentration had the lowest overall goblet cell count (Table 2 and Figure 5).

When we compared all 2,4-D-exposed animals, regardless of concentration, to the control group, a shift in mucus composition was observed, with a tendency for goblet cells to transition from neutral to acidic mucus. Additionally, there was a slight reduction in the total goblet cells count in 2,4-D-exposed animals (*p* < 0.05) (Figure 5E).

Exposed animals showed a small difference in goblet cell count compared to the control ones (Cohen’s d = 0.403141). However, the medium-concentration group exhibited very large differences when compared to the control (d = 2.253189) as well as to the low (d = 2.487532) and high concentration 2,4-D groups (d = 1.476252).

The lower number of goblet cells and the change in mucus pattern to acidic shows that 2,4-D damages these cells, which impacts the respiratory tract’s protective mechanism.

#### 3.1.5. Analysis of the Presence of Mucus in Goblet Cells

Not only the number of goblet cells and the pattern of mucus produced, but also the amount of mucus produced can impact the respiratory tract’s protective mechanism [11].

**Figure 6:**
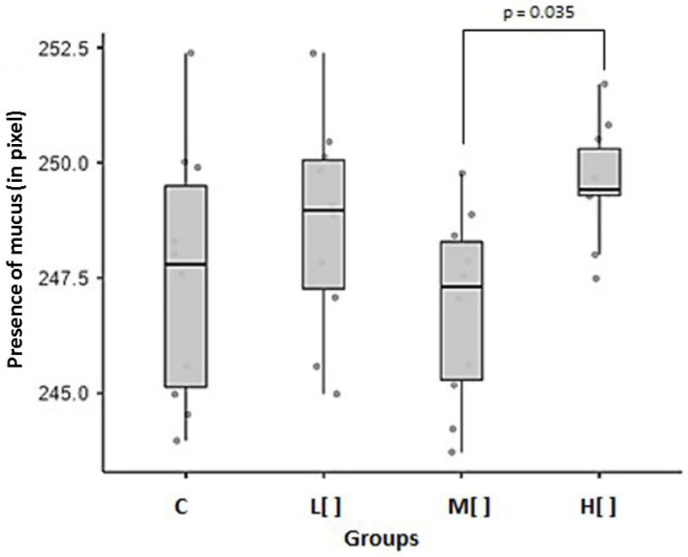
Mean and standard error of the presence of mucus in goblet cells (in pixels) by study group (*n* = 40). C: control group; L[ ]—group exposed to a low concentration of 2,4-D; M[ ]—group exposed to a medium concentration of 2,4-D; H[ ]—group exposed to a high concentration of 2,4-D.

Animals in the high concentration 2,4-D group showed a statistically significant difference in the presence of mucus in goblet cells compared to those in medium concentration group (*p* = 0.035) (Figure 6).

Exposed animals showed a small difference in the presence of mucus compared to the control group (Cohen’s d = 0.326426). However, the high-concentration group exhibited a large difference compared to the control group (d = 0.955056), a moderate difference compared to the low-concentration group (d = 0.516697), and a very large difference compared to the medium-concentration group (d = 1.61386). Additionally, the low-concentration group showed a large difference from the medium-concentration group (d = 0.825626).

2,4-D did not interfere with the presence of mucus in the cells, except in animals that were exposed to low concentrations of this herbicide, possibly because this group had a lower number of goblet cells.

#### 3.1.6. NORs Count

Nucleolar organizing regions (NORs) are tandemly repeated sequences of ribosomal genes (rDNA). The ability of these proteins to be stained with silver nitrate is because some proteins associated with NORs have acidic domains that contribute to silver uptake. Silver-stained proteins are nonhistone intranucleolar proteins that are linked with transcriptionally active sites of rDNA, as observed in metaphase and interphase cells. Thus, NORs can be considered markers of protein synthesis and the cell proliferation rate [21,22].

**Figure 7:**
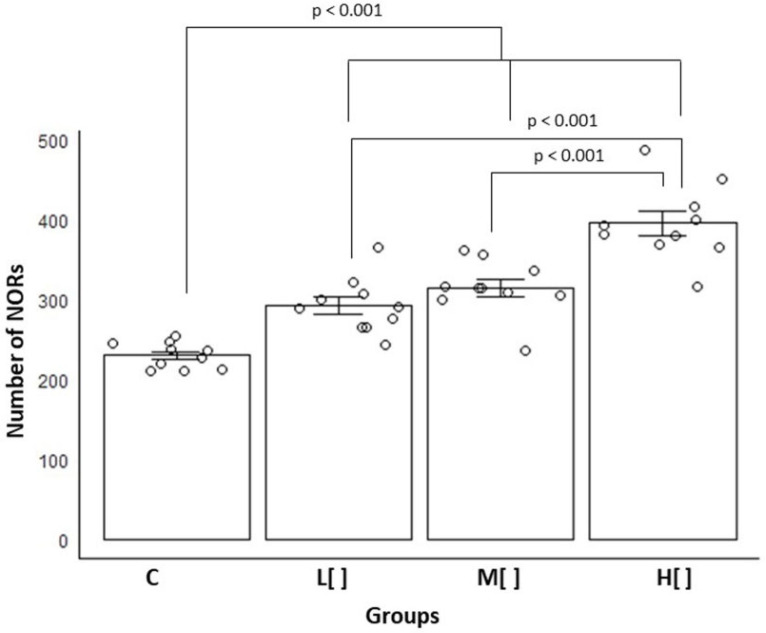
Means and standard errors of the number of NORs in tracheal epithelial cells by study group (*n* = 40). C: control group; L[ ]—group exposed to a low concentration of 2,4-D; M[ ]—group exposed to a medium concentration of 2,4-D; H[ ]—group exposed to a high concentration of 2,4-D.

The number of NORs in tracheal epithelial cells increased as the concentration of 2,4-D rose (Figure 7). Among all groups, the animals in the highest concentration group exhibited the highest NOR count, with an average of 396.1 (SD = 44.78) (*p* < 0.05).

Exposed animals showed a very large difference in NOR count compared to control animals (Cohen’s d = 2.462341). The high-concentration group exhibited very large differences when compared to the control (d = 4.950008) as well as to the low- and medium-concentration groups (d = 2.646532 and d = 2.055981, respectively). Additionally, the medium-concentration group showed a moderate difference from the low-concentration group (d = 0.696491).

The progressive increase in NORs as the concentration of exposure to 2,4-D increased shows that cells exposed to this herbicide are exhibiting greater cellular activity due to increased protein synthesis or proliferation.

### 3.2. Correlation Analysis

Correlation analysis is used to measure and quantify the strength and direction of the linear association between two or more variables, verifying whether changes in one are associated with changes in another. It helps to understand how factors influence each other [23].

**Figure 8:**
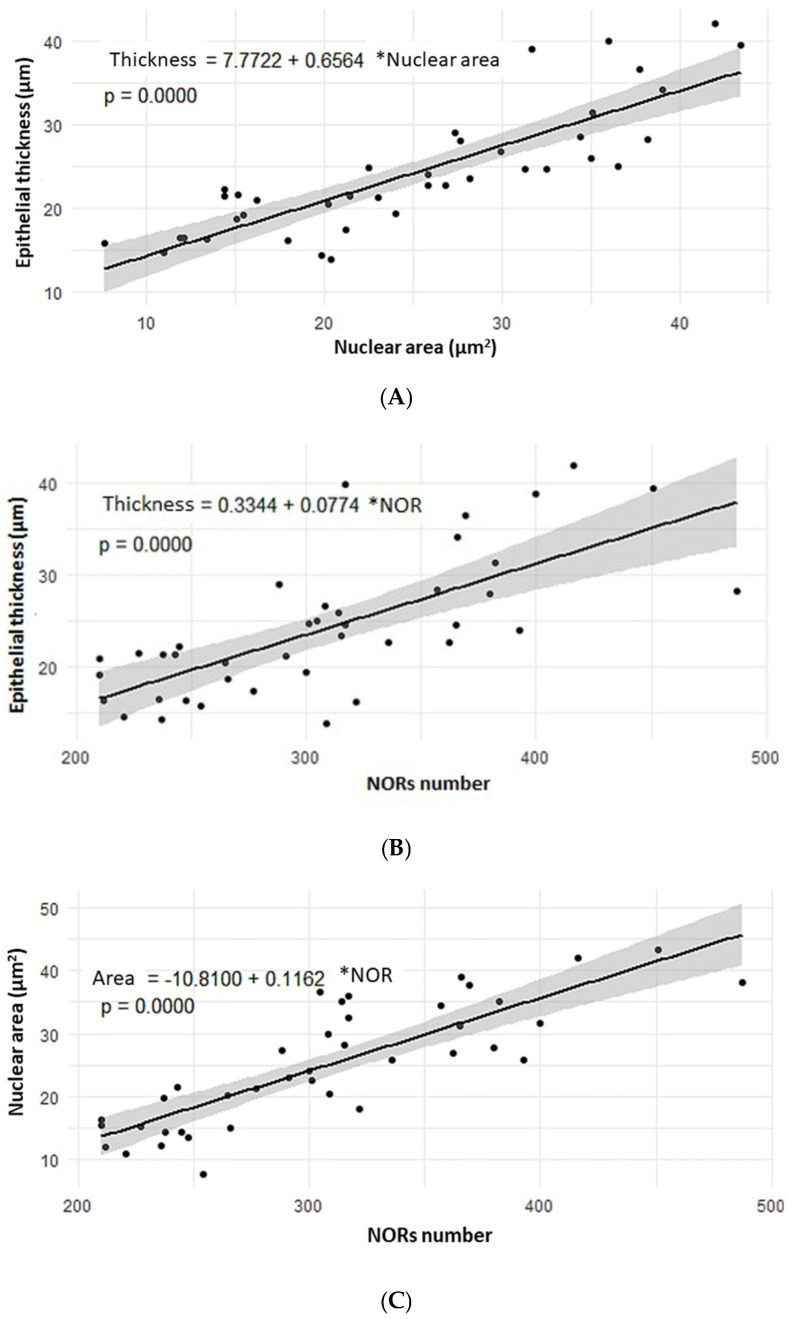
Scatter plots. (**A**) Correlation between epithelial thickness (in µm) and nuclear area (in µm^2^) (Pearson’s R= 0.8416—*p* < 0.00001). (**B**) Correlation between epithelial thickness (in µm) and NOR count (Pearson’s R= 0.7089—*p* < 0.00001). (**C**) Correlation between the nuclear area (in µm^2^) and NOR count (Pearson’s R= 0.8303—*p* < 0.00001).

A positive and strong correlation was observed between epithelial thickness and nucleus area (R = 0.8416; *p* < 0.00001), as well as between epithelial thickness and the NOR score (R = 0.7089; *p* < 0.00001). Additionally, a positive and strong correlation was also found between nucleus area and the NOR score (R = 0.8303; *p* < 0.00001) (Figure 8).

The strong positive correlation between the evaluated parameters, where an increase in one leads to an increase in the other, indicates that the observed changes are adaptive to the tissue damage caused by 2,4-D. The higher number of NORs is a consequence of greater nuclear activity and thus a larger nuclear area, and both lead to an increase in cell size and thus in epithelial thickness.

## 4. Discussion

This is the first study to evaluate and demonstrate the histological damage of the tracheal epithelium associated with chronic inhalation exposure to the herbicide 2,4-D at concentrations commonly used in crops.

Exposure to 2,4-D led to a higher number of mast cells in animals subjected to low concentrations. Regardless of the exposure level, it also caused an increase in tracheal epithelial thickness, the nuclear area of epithelial cells, and the NORs count. Additionally, the mucus composition in goblet cells showed a shift from neutral to acidic, accompanied by a reduction in the overall goblet cell count in exposed animals. These adaptive changes show that the herbicide 2,4-D damages the respiratory epithelium. They may compromise the protective mechanisms of the respiratory epithelium, and thus, the functioning of the respiratory system as a whole and not just the airways.

Pesticides have been widely adopted in agriculture since the end of World War II, when the chemical weapon industry found a new application for their products [24]. Those who work directly with pesticides, such as applicators and workers who do not adhere to reentry periods and do not use personal protective equipment, constitute a group at greater risk of poisoning [9,24].

Previous studies have shown that 2,4-D can induce inflammatory process in the lung epithelium, marked by an increase in lymphomononuclear cells [25]. However, in our study, inhalation of 2,4-D did not lead to a higher incidence of tracheal epithelial inflammation in exposed animals compared to the control. Notably, intense inflammation was noted only in the high concentration group. The inflammatory response to 2,4-D exposure may be triggered by the release of proinflammatory cytokines and the activation of oxidative stress pathways, both of which are commonly linked to exposure to environmental toxins [26]. This mechanism could explain the heightened inflammation observed in animals subjected to high concentrations of the 2,4-D.

Metaplasia requires continuous and intense stimuli to establish it [27]. Chronic inflammation is frequently linked to adaptive cellular changes, such as metaplasia, which occur as a response to harmful stimuli [19]. There was no increase in the occurrence of squamous metaplasia in the tracheal epithelium of animals exposed to 2,4-D at the concentrations and durations examined. This is likely due to the fact that the chronic inflammation observed was mild in most cases, and there was no significant rise in the incidence of inflammation in animals exposed to 2,4-D. These data suggest that the exposure was not sufficient to induce such severe tissue damage that would lead to this adaptive change.

In addition, no cases of dysplasia or neoplasia were found in animals of the 2,4-D groups, suggesting that the herbicide was not capable of inducing significant genetic alterations in the tracheal epithelia that would result in neoplastic transformation. These findings are consistent with previous studies that found no evidence that 2,4-D is carcinogenic in long-term studies in rodents, which is reinforced by the lack of evidence of genotoxicity or mutagenicity of 2,4-D in in vitro and in vivo studies [6,8,10].

Mast cells are involved in both the regulation of allergic reactions and the control of inflammatory processes. They express the high-affinity IgE receptor (FcεRI), which has a high affinity for immunoglobulin (Ig) E. When IgE bound to mast cells binds to an antigen, it promotes the release of inflammatory mediators, such as histamine, heparin, and cytokines, resulting in allergic reactions such as asthma. Furthermore, mast cells play important roles in chronic inflammation and tissue repair by releasing mediators that stimulate fibroblast growth, collagen production and tissue healing [28,29].

With respect to respiratory tract toxicity, Fukuyama et al. [30] reported that 2,4-D induced immediate respiratory allergic reactions, with increased IgE, eosinophils, neutrophils, and chemokines in bronchoalveolar lavage fluid after endotracheal instillation of 2,4-D in BALB/c mice. An in vitro study revealed that compounds such as methoxychlor, an organochlorine pesticide (the same class of 2,4-D), induce increased mast cell degranulation and intensify IgE-mediated allergic responses. These actions occur through the regulation of signal transduction mediated by FcεRI, which involves the activation of signaling cascades such as Syk and PLCg1/2. Therefore, investigating whether the herbicide 2,4-D can influence these signaling processes and mast cell degranulation is pertinent [31].

In the present study, animals in the low concentration group exhibited the highest mast cells count in the tracheal epithelium, indicating a more pronounced inflammatory and allergic response. This rise in mast cell count could be attributed to the initial activation of the immune system in response to 2,4-D, similar to findings in studies involving the organochlorine methoxychlor [31]. Conversely, animals in the medium and high concentration groups showed fewer mast cells, which may suggest a suppression of mast cell activation or recruitment, potentially due to desensitization of the tracheal epithelium [19,32]. Studies comparing mast cell counts with serum IgE levels may help clarify the role of mast cells in the tracheal epithelium following inhalation exposure to 2,4-D.

In two previous studies by our group assessing oral damage from 2,4-D exposure (at the same concentrations as in the current one), a decrease in mast cell number was observed in the tongues of mice acutely exposed by inhalation, independent of the 2,4-D concentration [33]. This was in contrast to chronic exposure, where higher mast cell count were found in rats exposed to both low and high concentrations of 2,4-D [34]. The effects of 2,4-D on the immune systems of humans and rodents are debated, with some studies indicating immune stimulation [1,35] while others suggest immunosuppression [1,36,37]. These effects seem to depend on the concentration and formulation of the 2,4-D-based product, as well as the organ affected and the duration of exposure, as our studies suggest.

The increase in tracheal epithelial thickness and nuclear area reflects a tissue reaction to chronic stress and tissue damage that occurred mainly through exposure to high concentrations of 2,4-D. Thus, the increased epithelial thickness suggests reactive hypertrophy, which involves an increase in cellular components and functions, expanding cell volume as an attempt by the tissue to intensify the physical barrier against the aggressive agent [19]. Moreover, the enlargement of the nuclear area indicates a proliferative response with increased DNA and protein synthesis, indicating possible genotoxic stress. Both phenomena are manifestations of a cellular response to damage, evidencing that the tissue is in a state of regeneration [19,38].

In this study, we noted a gradual rise in the number of NORs as the concentration of 2,4-D increased. A similar pattern of increased NORs linked to 2,4-D concentration was also observed in earlier studies conducted by our group, including acute exposure in mice [33] and chronic exposure in rats [34]. Also, the rise in NORs count was correlated with increases in the thickness and nuclei area of the tracheal epithelium. These data reveal that 2,4-D causes cellular and nuclear activity in response to damage/aggression.

The lower count of goblet cells in the medium concentration of 2,4-D group, combined with the tendency for the mucus pattern to revert to acid in the animals exposed to 2,4-D, raises questions about the selective toxicity of the pesticide. Goblet cells play a key role in mucus production, so the decrease in these cells likely indicates direct damage or cell death caused by 2,4-D. Additionally, the change in the mucus pattern may signal an early adaptation of the remaining cells to an inflammatory environment [38]. Changes in mucus composition, such as an alteration in the balance between neutral and acidic mucins, result in mucus that is thick and poorly hydrated. This change can compromise the ability of airways to clear mucus, leading to chronic respiratory problems. In advanced stages, the goblet cells loss can predispose the epithelium to additional infections and injuries [39]. These data, associated with an increase in nuclear area, epithelial thickness, and NORs count and the absence of an increased incidence of squamous metaplasia, show that the herbicide 2,4-D causes cellular damage; however, the damage is not so intense that the organism cannot recover or needs to adapt.

The difference in the presence of mucus in goblet cells between the high- and medium-concentration groups could be attributed to the lower count of goblet cells in animals in the medium concentration group. The greater mucus production by animals of the high concentration group appears to be closely linked to the hypertrophy process that the tissue underwent [38].

The quality of mucus and its effectiveness in protecting the tracheal epithelium may be compromised, especially if the composition of the mucus is altered, as suggested by the tendency of the mucus pattern to revert to acidic. In the long term, this compensation may be insufficient, leading to more serious dysfunctions, such as acute and chronic tracheobronchitis, and an increased risk of pneumonia [19,38,39].

Structural changes do not always lead to functional changes and vice versa. Studies that evaluate mucociliary transport speed or ciliary beat frequency through inhalation exposure to 2,4-D may better establish whether the observed histological changes can result in functional alterations.

Future studies that assess histological changes in conjunction with biochemical and functional alterations and markers of oxidative and inflammatory stress may help to elucidate the mechanism of action of the herbicide 2,4-D on the respiratory epithelium. Similarly, an assessment of a period longer than six months of exposure may show whether the organism can actually recover from the aggression triggered by the herbicide to the trachea.

## 5. Conclusions

This study demonstrated that chronic exposure to 2,4-D, under the parameters used (concentrations and exposure time), induces reactive histopathological alterations in the tracheal epithelium of rats, characterized by increases in the epithelial thickness, in the nuclear area of the cells and in the NORs count. Furthermore, low concentrations of 2,4-D may cause an allergic response in the tracheal epithelium, whereas exposure to higher concentrations may have a desensitizing effect on trachea. The tendency for a reduction in goblet cells in exposed animals and the alteration in the pattern of mucus production to acid indicate that 2,4-D may compromise mucociliary function and predispose the epithelium to additional lesions over time.

Therefore, the use of personal respiratory protective equipment during the management and use of 2,4-D is essential to prevent possible damage caused by this herbicide to the respiratory tract.

## Figures and Tables

**Table 1:** Incidence of inflammation (according to its intensity) and squamous metaplasia in the tracheal epithelium, according to the study group (*n* = 40).

Groups	Inflammation			Metaplasia
	Mild	Moderate	Intense	
Control	4/10 ^a^	2/10 ^a^	0/10 ^a^	2/10 ^a^
Low 2,4-D concentration	7/10 ^a^	2/10 ^a^	0/10 ^a^	2/10 ^a^
Medium 2,4-D concentration	6/10 ^a^	2/10 ^a^	0/10 ^a^	2/10 ^a^
High 2,4-D concentration	7/10 ^a^	2/10 ^a^	1/10 ^a^	3/10 ^a^

Lowercase letters compare groups at the same time and in the same column.

**Table 2:** Mean (standard deviation) of the number of goblet cells (per mm^2^), according to the mucus pattern and the study group (*n* = 40).

Groups	Neutral Mucus	Acidic Mucus	Total
Control	408.30 (±47.75) ^a^	239.0 (±46.12) ^a^	647.30 (±48.89) ^a^
Low 2,4-D concentration	312.50 (±37.74) ^b^	373.60 (±62.11) ^b^	686.10 (±70.79) ^a^
Medium 2,4-D concentration	235.60 (±37.18) ^c^	269.90 (±61.73) ^a^	505.50 (±74.37) ^b^
High 2,4-D concentration	334.10 (±56.40) ^b^	309.60 (±63.63) ^a,b^	643.70 (±109.53) ^a^

Lowercase letters compare groups at the same time and in the same column. Different lowercase letters: *p* < 0.05.

## Data Availability

All data generated or analyzed during this study are included in this published article.

## Abbreviations

The following abbreviations are used in this manuscript:

2,4-D	2,4-dichlorophenoxyacetic acid
ANOVA	analysis of variance
ANVISA	Agência Nacional de Vigilância Sanitária/Brazilian National Health Surveillance Agency
g.a.i./ha	grams of active ingredient per hectare
H[ ]	2,4-D high-concentration
HE	Hematoxylin–eosin
IARC	International Agency for Research on Cancer
Ig	immunoglobulin
L[ ]	2,4-D low-concentration
M[ ]	2,4-D medium-concentration
NIH	United States National Institute of Health
NORs	nucleolar organizing regions
PAS	Periodic acid–Schiff
rDNA	ribosomal genes
UNOESTE	Universidade do Oeste Paulista
